# Changes in the Peripheral Chemosensory System Drive Adaptive Shifts in Food Preferences in Insects

**DOI:** 10.3389/fncel.2018.00281

**Published:** 2018-08-29

**Authors:** Ayako Wada-Katsumata, Hugh M. Robertson, Jules Silverman, Coby Schal

**Affiliations:** ^1^Department of Entomology and Plant Pathology, North Carolina State University, Raleigh, NC, United States; ^2^Department of Entomology, University of Illinois at Urbana–Champaign, Urbana, IL, United States

**Keywords:** chemoreception, gustation, sensilla, nutrient sensing, glucose-aversion

## Abstract

A key challenge in understanding the evolution of animal behaviors is to identify cellular and molecular mechanisms that underlie the evolution of adaptive traits and behaviors in polymorphic populations under local selection pressures. Despite recent advances in fish, mice, and insects, there are still only a few compelling examples of major genes and cellular mechanisms associated with complex behavioral changes. Shifts in food or host preferences in insects, accompanied by changes in the peripheral chemosensory system, offer some of the best examples of adaptive behavioral evolution. A remarkable example is the German cockroach, *Blattella germanica*, a major indoor pest with a highly diverse omnivorous diet. Strong and persistent selection pressure with toxic-baits has induced rapid evolution of behavioral resistance in multiple cockroach populations. While typical cockroaches detect and accept the sugar glucose as a feeding-stimulant, behaviorally resistant cockroaches avoid eating glucose-containing toxic baits by sensing glucose as a deterrent. We review the peripheral gustatory neural mechanisms of glucose-aversion and discuss how the rapid emergence of taste polymorphisms can impede pest control efforts and affect foraging and mate-choice in adapted cockroach populations.

## Strong Anthropogenic Selection Drives Rapid Evolution of Pest Insects

Local selection pressures and genetic drift can lead to slow evolutionary divergence of allopatric populations; selection may be imposed by a wide variety of factors such as climate, resource availability, predation, and competition. It is often difficult, however, to disentangle how genetic traits respond to selection and lead to the evolution of adaptive behaviors in wild animals, because most behaviors are driven by multiple genes, and the evolutionary process is slow, requiring long-term observations. Anthropogenic selection, on the other hand, can impose much stronger evolutionary pressures over a shorter timeframe, often on genetically closed populations, leading to the rapid evolution of adaptive responses. Quintessential examples are the rapid evolution of resistance to antibiotics in medically important pathogens and to pesticides in agricultural pests, in response to human-imposed strong selection pressures (e.g., Gould et al., 2018).

The German cockroach, *Blattella germanica*, is a cosmopolitan synanthropic pest, living obligatorily in human environments such as homes, food processing facilities, restaurants, hospitals, transportation systems, and even farm buildings, among others (Schal, 2011). Cockroach control indoors is particularly challenging because of their close proximity to humans and pets, but in the early 1980s insecticide baits became a popular and highly effective strategy to control cockroach infestations. Because cockroaches must feed in preparation for nymphal development and adult reproduction, this requirement, coupled with their chewing mouthparts, made baits highly effective and ecologically safer than spray insecticides. Baits combine an insecticide with various phagostimulants, typically corn syrup composed of glucose and fructose in early bait formulations. However, within just a few years, bait performance was severely compromised as they rapidly induced physiological and behavioral resistance in cockroach populations (Silverman and Bieman, 1993; Wang et al., 2004). Most interesting among these was a population collected in Florida in 1989 that behaviorally shunned toxic baits; these field-collected cockroaches rejected glucose, a phagostimulant and nutrient ingredient in baits, but they had no metabolic resistance to the insecticide in the bait (Silverman and Bieman, 1993). This trait rendered all glucose-containing baits ineffective against these glucose-averse (GA) cockroaches (Silverman and Liang, 1999), and in response, bait manufacturers promptly reformulated bait products at considerable costs.

**FIGURE 1:**
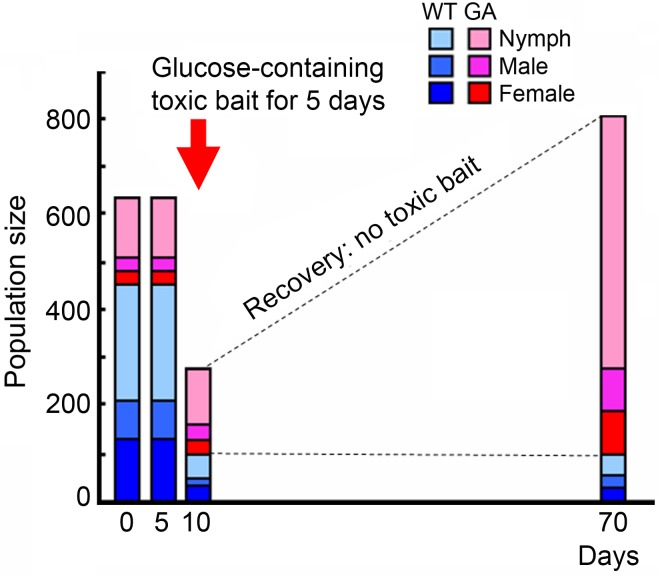
Population replacement from WT to GA cockroaches after exposure to glucose-containing toxic bait for 5 days (figure was modified from Wada-Katsumata et al., 2014, with permission from The Japanese Society for Comparative Physiology and Biochemistry). Glucose-containing toxic bait differentially killed WT cockroaches, favoring the survival of GA cockroaches.

The GA trait is heritable, controlled by a single major gene that follows Mendelian inheritance patterns (Silverman and Bieman, 1993): Crosses of homozygous wild-type (WT) and homozygous GA cockroaches result in 100% glucose-rejecting cockroaches, and in the F1 cross the GA trait is expressed in 75% of the progeny (50% heterozygous GA, 25% homozygous GA), while 25% are homozygous WT that never reject glucose at any concentration (Wada-Katsumata et al., 2014). Phenotyping of GA cockroaches is uncomplicated because homozygous GA individuals have 10-fold greater deterrence for glucose than heterozygous GA cockroaches (Silverman, 1995). Importantly, injection of high concentrations of glucose into the hemocoel did not adversely affect the physiology or behavior, including feeding preference for glucose, foraging, and sexual maturation of GA and WT cockroaches. These findings indicate that glucose-aversion is mediated by information processing via the chemosensory system (Wada-Katsumata et al., 2011) and not through toxic effects associated with glucose. Glucose-aversion thus confers an enormous advantage in the presence of toxic baits due to behavioral rejection of these pesticide-containing products. Recent studies indicate that even short-term selection with a glucose-containing toxic bait can rapidly increase the frequency of GA cockroaches (Wada-Katsumata et al., 2014; **Figure 1**). This trait is now common in multiple field populations (Wang et al., 2004; Wada-Katsumata et al., 2013). Importantly, because GA cockroaches consume less glucose-containing diet, this trait is maladaptive in bait-free environments as GA cockroaches must seek glucose-free foods. Two decades after its discovery, the neural basis of this fascinating taste polymorphism was demonstrated with electrophysiological studies (Wada-Katsumata et al., 2011, 2013), but its molecular mechanism remains to be determined.

## The Insect Gustatory System

The ability to detect and discriminate tastants is essential because tastants convey important information about the quality and nutritional value of food, allowing animals to avoid potentially toxic or spoiled food, and in some instances guiding mate-choice decisions. As in humans and mice, the peripheral gustatory system of insects is mainly localized in the mouthparts, but other appendages such as the antennae, feet and wings may be involved as well. Detection and assessment of the quality and intensity of tastants occurs in bipolar Gustatory Receptor Neurons (GRNs) whose cell bodies and dendrites are housed within hair-like cuticle-lined sensilla with a pore at the tip (Newland et al., 2009). The organization of GRNs has been well described in the common fruit fly *Drosophila melanogaster*. The labellum contains approximately 31 sensilla which are grouped into morphological classes based on their length. Each sensillum contains two or four different types of GRNs, with each GRN expressing a specific taste modality. Therefore, GRNs are denoted sweet-, bitter-, water-, or salt-GRNs. While water- and salt-GRNs express Ionotropic Receptors (IRs) and pickpocket (PPK) receptors, sweet- and bitter-GRNs are characterized by combinatorial sets of co-expressed Gustatory Receptors (GRs) which recognize particular tastant molecules (Montell, 2009; Freeman and Dahanukar, 2015; Scott, 2018). For example, *Drosophila* has 68 GRs, and sweet-GRNs express members of a conserved clade of sugar GRs. Bitter-GRNs, on the other hand, are tuned to aversive tastants such as noxious substances and are characterized by subsets of GRs that never overlap with GRs expressed in sweet-GRNs. Both sugar and bitter receptors are thought to be composed of multimeric GRs (Montell, 2009; Freeman and Dahanukar, 2015; Scott, 2018). The axons of GRNs with the same modal specificity (taste quality) project directly to the same region in the central nervous system (CNS). Quality, strength and duration of stimuli are represented as neuronal impulses by GRNs (Scott, 2018). The sweet-GRNs mediate appetitive behavior via CNS processing, whereas the responses of bitter-GRNs mediate rejection behavior. Therefore, modifications in tastant discrimination by GRs and GRNs, which represent the peripheral first stage in gustatory information processing, can critically impact the expression of gustatory behavior.

## Gustatory System of the German Cockroach and Glucose-Aversion

**FIGURE 2:**
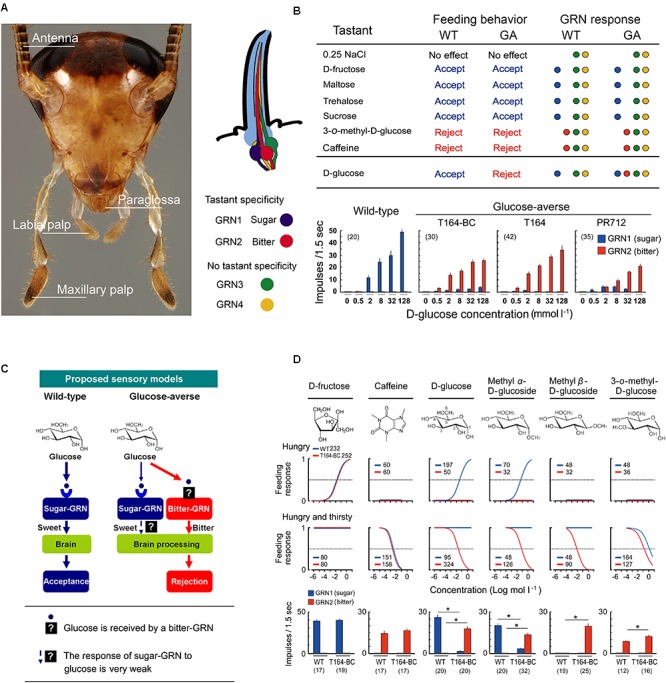
Gustatory neural mechanism of glucose-aversion (figures are adapted from Wada-Katsumata et al., 2013, with permission from The American Association for the Advancement of Science). **(A)** Head of male German cockroach, *Blattella germanica*, showing the four sensory appendages (antennae, maxillary palps, labial palps, paraglossae) and schematic of a sensillum containing four gustatory receptor neurons (GRNs). **(B)** Summary of behavioral and GRN sensitivities of wild-type (WT) and GA (T164-Backcross) cockroaches to various tastants, and dose-GRN responses to glucose in a WT and three GA cockroach populations (T164-backcross with WT, T164, field-collected PR712 strain). Bitter-GRNs of GA cockroaches respond to glucose, whereas in WT cockroaches the bitter-GRN never responds to glucose. **(C)** Proposed model of glucose-aversion in the German cockroach. Glucose-aversion could be encoded by changes in glucose detection of bitter-GRNs of the paraglossa. Feeding responses in animals can be altered by genetic polymorphisms in gustatory receptors (GRs) over a finite range from highly sensitive to completely insensitive to a particular tastant. However, in bait-selected cockroach populations, the modal specificity of glucose, rather than sensitivity to glucose, has been strikingly transformed from “sweet” and highly phagostimulatory to “bitter” and highly deterrent. And these changes occurred at the sensory level. Generally, insect bitter-GRNs co-express a large number of gustatory receptors (GRs) and are broadly tuned to detect various deterrents. The co-expression of GRs accounts for the unique sensitivity of bitter-GRNs and their capacity to selectively respond to deterrents. Our studies suggest two major hypotheses: (a) Modifications of the structure of GRs on the bitter-GRN cause acceptance of glucose; (b) The mis-expression of native glucose GRs on the bitter-GRN result in glucose acceptance. Recruiting glucose as a bitter-GRN ligand expresses glucose-aversion as a novel adaptive behavior that offers protection against toxic baits. **(D)** Structure-activity experiments. Top, Chemical structures of phagostimulants and detterents. Middle, Dose-feeding responses of WT (blue), and GA (red, T164-Backcross) cockroaches. Hungry, cockroaches were motivated to accept phagostimulants but not water; Hungry and thirsty, cockroaches were motivated to take both phagostimulants and water. Feeding response is shown as the proportion of cockroaches ingesting the test solution. Legends indicate sample size. GA cockroaches rejected all tested compouds except fructose. Bottom, The sugar and bitter-GRNs of WT and GA cockroaches respond differentially to six compounds. Number in parentheses indicates tested sensilla. (^∗^*P* < 0.05, Student’s *t*-test).

GRNs of the German cockroach are housed in sensilla on the mouthparts and antennae (Wada-Katsumata et al., 2009, 2011, 2013). While foraging, cockroaches discriminate food sources first with the antennae, the most distal appendages from the mouthparts, then with the maxillary and labial palps, and finally with the paraglossae, the gateway chemosensory structure to the mouth (**Figure 2A**); each of these can evaluate and discriminate nutrients from noxious substances. Adult females possess 2,380 gustatory sensilla (sensilla chaetica B) on the antenna, and each antenna of adult males house 2,360 gustatory sensilla (Ramaswamy and Gupta, 1981). The numbers and topologies of gustatory sensilla in the maxillary and labial palps are largely unknown. Behavioral assays with cockroaches whose taste organs were systematically ablated showed that differential inputs from these four sensory appendages (**Figure 2A**) mediate appetitive and aversive responses (Wada-Katsumata et al., 2011). While all four sensory appendages can stimulate acceptance and rejection of tastants, the paraglossae alone represent a minimal system for further investigations. They are the last checkpoint before ingestion and the paraglossae have the highest sensitivity to phagostimulants and deterrents (Wada-Katsumata et al., 2011).

Each paraglossa contains approximately 60 gustatory sensilla with no sexual dimorphism (Wada-Katsumata et al., 2009). At least four types of GRNs are housed in each sensillum (**Figure 2A**). Two GRNs have ligand specificity and are denoted sweet-GRN and bitter-GRN. The other two GRNs have no ligand specificity and are involved in sensing osmolality. Positive correlations among feeding responses, GRN chemosensation and the concentration of tastants suggest that chemosensation of the sweet-GRNs contributes to appetitive feeding responses to nutrient sugars such as glucose, fructose, sucrose, maltose, maltotriose, and trehalose. Bitter-GRNs contribute to aversive feeding responses to noxious substances such as caffeine (Wada-Katsumata et al., 2013).

Glucose-aversion could result from heritable changes in the processing of chemosensory cues in the peripheral sensilla or in the CNS. Comparative electrophysiological analyses of GRN sensitivities to various tastants in homozygous WT, homozygous GA (Silverman and Bieman, 1993), backcrosses of WT and GA, and two field-collected GA populations revealed that polymorphisms in GRN sensitivity drove glucose-aversion (Wada-Katsumata et al., 2013). Tip recordings from gustatory sensilla (Hodgson et al., 1995; Wada-Katsumata et al., 2009) revealed that in both WT and GA cockroaches phagostimulants (e.g., fructose) stimulated a sweet-GRN and guided appetitive behavior, whereas deterrents (e.g., caffeine) stimulated a bitter-GRN and drove aversive behavior (**Figure 2B**; Wada-Katsumata et al., 2013). Glucose, like fructose, also simulated the sweet-GRN in WT cockroaches, but in GA cockroaches, glucose stimulated both sweet- and bitter-GRNs. Three key features of the GRN responses are that: (a) the bitter-GRN responds to glucose in a concentration-dependent manner, (b) the electrophysiological responses of the sweet-GRN to glucose are greatly attenuated in GA cockroaches, and (c) the bitter-GRN response correlates with aversive behavior. This relationship between stimuli, electrophysiological responses, and behavioral responses suggests that the bitter-GRN acquired sensitivity to glucose, and this change is responsible for glucose-driven aversions.

These results suggest two major hypotheses, although others are possible as well. Mutations caused: (a) structural modification(s) of GRs in the bitter-GRN that enable detection of glucose, and/or (b) misexpression of native glucose GRs in the bitter-GRN (**Figure 2C**). Structure-activity studies using glucose-derivatives revealed that in WT cockroaches glucose and methyl α-D-glucose stimulated the sweet-GRNs and appetitive feeding behavior. Methyl β-D-glucose did not stimulate either sweet- or bitter-GRNs and did not elicit a feeding response (**Figure 2D**; Wada-Katsumata et al., 2013). This indicates that sweet-GRNs of WT cockroaches have binding sites for glucose and methyl α-D-glucose, and that both sweet- and bitter-GRNs have no binding sites for methyl β-D-glucose. On the other hand, in GA cockroaches, both glucose and its two methyl-derivatives stimulated the bitter-GRN and induced aversive feeding responses. In other words, bitter-GRNs of GA cockroaches have binding sites for methyl β-D-glucose and mediate aversive feeding behaviors to this compound. These results tentatively support the hypothesis that the glucose-sensitive GRs of the bitter-GRNs of GA cockroaches are differently tuned from the native glucose GRs on the sweet-GRNs. It is possible, however, that the ectopic expression of sugar GRs on the bitter GRNs was accompanied by modifications in their ligand affinities.

Our results show that a gain-of-function adaptation has emerged in the peripheral gustatory system. Namely, recognition of glucose by receptors on bitter-GRNs specifies glucose as a bitter tastant, changing its valence (taste quality) from sweet to bitter, and causing a novel adaptive behavior to emerge which protects the cockroach from the lethal effects of glucose-containing toxic baits. Moreover, the aversion to glucose is further amplified by a pre-existing intrasensillar inhibition of sweet-GRN responses by deterrents. The gain-of-function of glucose receptors in bitter GRNs fits with the dominant nature of the genetics of this trait. Thus, glucose-aversion is a compelling example of a chemosensory-based adaptation that conferred behavioral resistance to anthropogenic selection, protecting the German cockroach from insecticides.

## Molecular Mechanisms of Glucose-Aversion: Work in Progress

Genomic and bioinformatic analyses of GR organization in holometabolous insects, including Diptera (Clyne et al., 2000; Scott et al., 2001; Hill et al., 2002; Robertson et al., 2003; Kent et al., 2008), Coleoptera (McKenna et al., 2016), Lepidoptera (Guo et al., 2017), and Hymenoptera (Robertson and Wanner, 2006) characterized 68 putatively functional GRs in *D. melanogaster*, 76 in the African malaria mosquito (*Anopheles gambiae*), 91 in the yellow fever mosquito (*Aedes aegypti*), 222 in the red flour beetle (*Tribolium castaneum*), 234 in the Asian longhorned beetle (*Anoplophora glabripennis*), 76 in the silkmoth (*Bombyx mori*), 5 in the fig wasp (*Ceratosolen solmsi*), and 12 in the European honey bee (*Apis mellifera*). The *Drosophila* GRs are functionally categorized for CO_2_ detection, sugar and amino acid detection (sweet), noxious substance detection (bitter) and pheromone detection. These patterns also suggest that diet specialists with narrow host ranges have few GRs, whereas generalist herbivores and omnivores evolved more diverse GRs. Indeed, the GR organization of omnivorous cockroaches support this pattern: the American cockroach *Periplaneta americana* and the German cockroach genomes encode 522 and 545 putatively functional GRs, respectively (Harrison et al., 2018; Li et al., 2018; Robertson et al., 2018). These GRs fall into the general clades of bitter receptors, sugar receptors and CO_2_ receptors, but most of the GRs are likely involved in the detection of bitter tastants. The German cockroach has 14 sugar GR candidates (BgerGr1-14). BgerGr431 is a divergent gene and a relative of the fructose receptor DmelGr43a lineage. It is present in all neopteran insects examined, but not in the dampwood termite (*Zootermopsis nevadensis*) (Terrapon et al., 2014; Robertson et al., 2018). Our ongoing functional analysis of the GRs of the German cockroach is a first step toward understanding the molecular mechanisms of glucose-aversion. Although, there is the awareness that GRN response to tastants could be supported by not only GRs, but also by other chemosensory proteins, such as IRs, OBPs, PPKs, and TRP channels, functional analysis using RNAi knockdown of candidate sugar receptor genes of WT and GA cockroaches will demonstrate if the misexpression of sugar receptor genes in bitter-GRNs of GA cockroaches mediates glucose-aversion.

## Glucose-Aversion Modifies Foraging, Mating and Population Dynamics

Foraging of GA cockroaches is impacted by associative olfactory learning. While WT cockroaches learn to associate bait odors with reinforcement from glucose, GA cockroaches associate the bait odors with punishment from glucose and promptly learn to avoid the bait (Wada-Katsumata et al., 2016). In a learning paradigm, a complex chocolate food odor was innately preferred over the complex odor of vanilla by both WT and GA cockroaches, but GA cockroaches quickly learned to associate chocolate odor with glucose as punishment and subsequently avoided chocolate while foraging. The avoidance response was retained for 3 days after only 1 h of self-training. On the other hand, odors paired with glucose positively reinforced the food preferences of WT cockroaches. Associative learning and memory thus amplify the adaptive response to glucose-containing toxic baits and exacerbate pest control efforts. As well, however, GA cockroaches learn to avoid glucose-containing non-toxic foods, lowering their overall fitness in a bait-free environment.

Glucose-aversion can drive other behavioral polymorphisms in German cockroach populations, even in bait-free natural environments. The population dynamics of WT and GA is significantly impacted by a difference in mate-choice of WT and GA females (Jensen et al., 2017). There are no differences in nymph development and female fecundity between GA and WT cockroaches provisioned with glucose-free food. However, although WT and GA males do not preferentially court GA or WT females, WT females tend to accept WT males more than GA males. The GA females, on the other hand, mate equally with WT and GA males. A potential explanation for this assortative mating by females is that gustatory preferences affect courtship behavior. During courtship, the female mounts the male and evaluates his quality by tasting a nuptial secretion that he offers on his tergum (Gemeno and Schal, 2004; Wada-Katsumata et al., 2009); the secretion contains sugars, including maltose and maltotriose (Kugimiya et al., 2003), which stimulate the female’s sweet-GRNs (Wada-Katsumata et al., 2009). This nuptial gift evolved under sexual selection to lure the female and position her for copulation. The differential mating success observed in this study suggests that WT and GA males have evolved nuptial secretion components that suit the gustatory preferences of the respective females in their population. Our model is that as WT males increase the amount of glucose-related sugars in their nuptial secretion in response to WT female preferences, nuptial feeding by GA females on WT males is interrupted by the taste of glucose. This hypothesis, that the reversed modal specificity of glucose causes GA females to sense the nuptial gift components as deterrents, impeding the completion of the courtship sequence, will be tested by comparative chemical analysis of the nuptial secretions of WT and GA males.

The difference in mate preferences also impacts the demography of populations initiated with equal numbers of WT and GA females and males. When provisioned with glucose-containing food, the proportion of WT cockroaches increased over 12 months because of high mortality and delayed nymph development of GA cockroaches (Jensen et al., 2017). But even when provisioned with rodent chow or chow supplemented with fructose, the proportion of WT cockroaches increased gradually. These results suggest that population growth in GA cockroaches is slower than in WT populations, partly because of assortative mating preferences.

## Conclusion

Glucose-aversion is expressed as a reversal in the modal quality of glucose from sweet and appetitive to bitter and aversive. This gain-of-function change results from a modification of the peripheral gustatory system wherein glucose stimulates not only sweet-GRNs, but also bitter-GRNs. Glucose-aversion can profoundly affect food choice and other traits that are under sexual selection, resulting in changes in sexual communication, mate choice and population dynamics. Under natural conditions, this trait is maladaptive and probably maintained in heterozygotes as a low frequency gustatory polymorphism. However, under the strong anthropogenic selection of insecticide- and glucose-containing baits, this trait is highly adaptive and supports behavioral resistance to insecticidal products. The glucose-aversion trait of the German cockroach is a remarkable example of how the evolution of a single altered gustatory trait under anthropogenic selection can shape the foraging ecology, sexual communication and population dynamics of populations.

## Author Contributions

AW-K, HMR, JS, and CS wrote the manuscript.

## Conflict of Interest Statement

The authors declare that the research was conducted in the absence of any commercial or financial relationships that could be construed as a potential conflict of interest.

## References

[B1] Clyne P. J. Warr C. G. Carlson J. R. (2000). Candidate taste receptors in *Drosophila*. *Science* 287 1830–1834. 10.1126/science.287.5459.1830 10710312

[B2] Freeman E. G. Dahanukar A. (2015). Molecular neurobiology of *Drosophila* taste. *Curr. Opin. Neurobiol.* 34 140–148. 10.1016/j.conb.2015.06.001 26102453 PMC4577450

[B3] Gemeno C. Schal C. (2004). Sex pheromones of cockroaches. in *Advances in Insect Chemical Ecology* eds Cardé R. T. Millar J. (New York, NY: Cambridge University Press) 179–247.

[B4] Gould F. Brown Z. S. Kuzma J. (2018). Wicked evolution: can we address the sociobiological dilemma of pesticide resistance? *Science* 360 728–732. 10.1126/science.aar3780 29773742

[B5] Guo H. Cheng T. Chen Z. Jiang L. Guo Y. Liu J. (2017). Expression map of a complete set of gustatory receptor genes in chemosensory organs of *Bombyx mori*. *Insect Biochem. Mol. Biol.* 82 74–82. 10.1016/j.ibmb.2017.02.001 28185941

[B6] Harrison M. C. Jongepier E. Robertson H. M. Arning N. Bitard-Feildel T. Chao H. (2018). Hemimetabolous genomes reveal molecular basis of termite eusociality. *Nat. Ecol. Evol.* 2 557–566. 10.1038/s41559-017-0459-1 29403074 PMC6482461

[B7] Hill C. A. Fox A. N. Pitts R. J. Kent L. B. Tan P. L. Chrystal M. A. (2002). G protein-coupled receptors in *Anopheles gambiae*. *Science* 298 176–178. 10.1126/science.1076196 12364795

[B8] Hodgson E. E. Lettvin J. Y. Roeder K. D. (1995). Physiology of a primary chemoreceptor unit. *Science* 122 417–418. 10.1126/science.122.3166.417-a 13246649

[B9] Jensen K. Wada-Katsumata A. Schal C. Silverman J. (2017). Persistence of a sugar-rejecting cockroach genotype under various dietary regimes. *Sci. Rep.* 7:46361. 10.1038/srep46361 28406167 PMC5390319

[B10] Kent L. B. Walden K. K. Robertson H. M. (2008). The Gr family of candidate gustatory and olfactory receptors in the yellow-fever mosquito *Aedes aegypti*. *Chem. Senses* 33 79–93. 10.1093/chemse/bjm067 17928357

[B11] Kugimiya S. Nishida R. Sakuma M. Kuwahara Y. (2003). Nutritional phagostimulants function as male courtship pheromone in the German cockroach. *Blattella germanica*. *Chemoecology* 13 169–175. 10.1007/s00049-003-0245-1

[B12] Li S. Zhu S. Jia Q. Yuan D. Ren C. Li K. (2018). The genomic and functional landscapes of developmental plasticity in the American cockroach. *Nat. Commun.* 9:1008. 10.1038/s41467-018-03281-1 29559629 PMC5861062

[B13] McKenna D. D. Scully E. D. Pauchet Y. Hoover K. Kirsch R. Geib S. M. (2016). Genome of the Asian longhorned beetle (*Anoplophora glabripennis*), a globally significant invasive species, reveals key functional and evolutionary innovations at the beetle-plant interface. *Genome Biol.* 17:227. 10.1186/s13059-016-1088-8 27832824 PMC5105290

[B14] Montell C. (2009). A taste of the *Drosophila* gustatory receptors. *Curr. Opin. Neurobiol.* 19 345–353. 10.1016/j.conb.2009.07.001 19660932 PMC2747619

[B15] Newland P. L. Cobb M. Marion-Poll F. (2009). *Insect Taste.* New York, NY: Taylor & Francis Group.

[B16] Ramaswamy S. B. Gupta P. (1981). Sensilla of the antennae and the labial and maxillary palps of *Blattella germanica* (L.) (Dictyoptera: Blattellidae): their classification and distribution. *J. Morphol.* 168 269–279. 10.1002/jmor.1051680303 30139196

[B17] Robertson H. M. Baits R. L. Walden K. K. O. Wada-Katsumata A. Schal C. (2018). Enormous expansion of the chemosensory gene repertoire in the omnivorous German cockroach *Blattella germanica*. *J. Exp. Zool. B Mol. Dev. Evol* 10.1002/jez.b.22797 [Epub ahead of print]. 29566459 PMC6175461

[B18] Robertson H. M. Wanner K. W. (2006). The chemoreceptor superfamily in the honey bee, *Apis mellifera*: expansion of the odorant, but not gustatory, receptor family. *Genome Res.* 16 1395–1403. 10.1101/gr.5057506 17065611 PMC1626641

[B19] Robertson H. M. Warr C. G. Carlson J. R. (2003). Molecular evolution of the insect chemoreceptor gene superfamily in *Drosophila melanogaster*. *Proc. Natl. Acad. Sci. U.S.A.* 100 14537–14542. 10.1073/pnas.2335847100 14608037 PMC304115

[B20] Schal C. (2011). “Cockroaches,” in *Handbook of Pest Control* ed. Moreland D. (Cleveland, OH: Mallis Handbook) 150–291.

[B21] Scott K. (2018). Gustatory processing in *Drosophila melanogaster*. *Annu. Rev. Entomol.* 63 15–30. 10.1146/annurev-ento-020117-043331 29324046

[B22] Scott K. Brady R. Jr. Cravchik A. Morozov P. Rzhetsky A. Zuker C. (2001). A chemosensory gene family encoding candidate gustatory and olfactory receptors in *Drosophila*. *Cell* 104 661–673. 10.1016/S0092-8674(01)00263-X 11257221

[B23] Silverman J. (1995). Effects of glucose-supplemented diets on food intake, nymphal development, and fecundity of glucose-averse, non-glucose-averse, and heterozygous strains of the German cockroach, *Blattella germanica*. *Entomol. Exp. Appl.* 76 7–14. 10.1111/j.1570-7458.1995.tb01941.x

[B24] Silverman J. Bieman D. N. (1993). Glucose aversion in the German cockroach. *Blattella germanica*. *J. Insect Physiol.* 39 925–933. 10.1016/0022-1910(93)90002-9

[B25] Silverman J. Liang D. (1999). Effect of fipronil on bait formulation-based aversion in the German cockroach (Dictyoptera: Blattellidae). *J. Econ. Entomol.* 92 886–889. 10.1093/jee/92.4.886

[B26] Terrapon N. Li C. Robertson H. M. Ji L. Meng X. Booth W. (2014). Molecular traces of alternative social organization in a termite genome. *Nat. Commun.* 5:3636. 10.1038/ncomms4636 24845553

[B27] Wada-Katsumata A. Ozaki M. Yokohari F. Nishikawa M. Nishida R. (2009). Behavioral and electrophysiological studies on the sexually biased synergism between oligosaccharides and phospholipids in gustatory perception of nuptial secretion by the German cockroach. *J. Insect Physiol.* 55 742–750. 10.1016/j.jinsphys.2009.04.014 19422830

[B28] Wada-Katsumata A. Silverman J. Schal C. (2011). Differential inputs from chemosensory appendages mediate feeding responses to glucose in wild-type and glucose-averse German cockroaches. *Blattella germanica*. *Chem. Senses* 36 589–600. 10.1093/chemse/bjr023 21467150

[B29] Wada-Katsumata A. Silverman J. Schal C. (2013). Changes in taste neurons support the emergence of an adaptive behavior in cockroaches. *Science* 340 972–975. 10.1126/science.1234854 23704571

[B30] Wada-Katsumata A. Silverman J. Schal C. (2014). Sugar aversion: a newly-acquired adaptive change in gustatory receptor neurons in the German cockroach. *Comp. Physiol. Biochem.* 31 220–230. 10.3330/hikakuseiriseika.31.220 23704571

[B31] Wada-Katsumata A. Silverman J. Schal C. (2016). Sugar-aversion: polymorphism of the peripheral gustatory system drives adaptive foraging behavior in the German cockroach. *Chem. Senses* 41:E117 10.1093/chemse/bjw091

[B32] Wang C. Scharf M. E. Bennett G. W. (2004). Behavioral and physiological resistance of the German cockroach to gel baits (Blattodea: Blattellidae). *J. Econ. Entomol.* 97 2067–2072. 10.1093/jee/97.6.2067 15666766

